# On weapons allometry and the form of sexual selection

**DOI:** 10.1098/rsos.251152

**Published:** 2025-09-24

**Authors:** Hironori Shinohara, Manmohan D. Sharma, Tanya M. Pennell, Kensuke Okada, David J. Hosken

**Affiliations:** ^1^ Center for Ecology and Conservation, University of Exeter, Cornwall Campus, Penryn, Cornwall, UK; ^2^ Faculty of Environmental, Life, Natural Science and Technology, Okayama University, Okayama, 700-8530, Japan

**Keywords:** inbreeding, selection, beetle, Gnatocerus

## Abstract

The study of trait scaling with body size (allometry) has a long history, and it has been argued that positive static allometry is an indicator of directional sexual selection. However, a range of allometries exists for sexually selected traits, and modelling shows this variation can be generated by altering the form of selection (fitness functions) on the trait and/or body size. Interestingly, in all models, positive allometry appears to emerge only when there is directional selection on trait size. Here, we report on a sexually selected trait that shows strong positive static allometry and yet appears to be under stabilizing selection. This surprising finding suggests the evolution of trait scaling is even more nuanced than currently appreciated.

## 1. Introduction

The study of trait scaling with body size has a long history [1–3], with the scaling of sexually selected traits—their allometry—still rather poorly understood [4]. Allometry can refer to three rather distinct phenomena: evolutionary patterns of scaling (across species), patterns of scaling during organismal development and the scaling of traits in individuals of the same age class (static allometry). This latter (static) allometry (hereafter allometry) is the focus of this study.

Trait scaling can be proportional, such that traits increase at the same rate as body size (isometry), or show negative or positive allometry. Negative allometry occurs when trait size increases with body size are less than proportional—the slope of the trait–body size relationship is less than one. This most notably occurs with metabolic rate [5,6], such that larger organisms have a lower relative metabolic rate than smaller organisms. Positive allometry, where trait size increases faster than body size (allometric slope >1), is notably found in traits subjected to sexual selection (e.g. [7–13]). This pattern is frequent enough that it has been suggested that positive allometry is a signal of sexual selection [9,14], and the positive allometry of sexually selected traits has been called one of the most common but poorly understood patterns in animal morphology [4]. Be that as it may, it is now clear that not all sexually selected traits show positive allometry [15,16], with male genital characters being one striking example (they tend to be negatively allometric: [17,18]). However, positive allometry in characters like weapons and signals has been linked to directional sexual selection favouring larger traits [15].

Theoretical exploration of allometric associations has shown that the shape of selection (e.g. directional/stabilizing) affects allometry, and a range of allometries can be generated with relatively minor changes in model fitness functions (e.g. [4,19]). For example, when traits are under directional selection, but also generate direct viability costs, negative allometry can evolve, while directional selection without the direct viability cost leads to isometry [19]. Additionally, in at least one empirical study where selection on traits has been directly estimated, directional selection is associated with negative trait allometry [20]. That is, directional selection need not generate positive allometry in theory or empirically. Interestingly, however, directional selection on trait size *is* required to generate positive allometry in trait-scaling models [4,14,19], and the sexual selection ‘positive allometry hypothesis’ is based on an assumption of directional selection for larger traits [15]. It has also been suggested that trait allometry can be used to infer the shape of selection acting on a trait (e.g. [10,11,16]). However, few studies assess allometry and estimate selection to directly assess whether all this holds water (but see, e.g. [20]).

Estimating selection on traits is, in principle, easy [21], but in practice can be more complicated [22]. This is because we can never measure the full phenotype and may therefore miss the precise traits under selection, and frequently, selection estimates are only made at one point in time [23], meaning we may miss the most important bouts of selection (see, e.g. [24]). Additionally, failure to detect selection now does not preclude selection acting on a trait in the past [25]. For all these reasons, inbreeding has been suggested as one way to estimate historical selection acting on phenotypes [26–29].

Inbreeding can be used to infer past selection because inbreeding depression moves traits towards low fitness values, which means towards selectively disfavoured values—inbreeding depression ‘points’ away from the direction of selectively favoured trait values [30–32]—and traits that show no inbreeding depression are either weakly linked to fitness or under stabilizing selection (i.e. mutations moving values up or down are selectively equivalent and hence there is no inbreeding depression) [32]. Inbreeding depression fundamentally results from directional dominance, and past selection explains the directionality of the dominance effect [32]. Basically, selection tends to remove dominant deleterious alleles and beneficial alleles should move towards fixation regardless of their degree of dominance. However, deleterious recessives, the effects of which are largely invisible to selection (they are masked in heterozygotes), are retained, and all this generates directionality to dominance effects (see [32] for a full discussion). Inbreeding exposes the effects of these deleterious recessive alleles and moves trait values away from high fitness. Thus, inbreeding depression shows the direction of past selection, and a lack of it is consistent with stabilizing selection if a trait is linked to fitness (table 1). And to reiterate, this approach has been used previously to infer historical selection [28,33]. To summarize, inbreeding depression is in the direction of low fitness and is the result of directional dominance due to past selection. As a result, it can be used to infer past selection acting on a trait, and this is likely to be especially useful for lab studies which are not able to fully recapitulate the ancestral/natural environment.

**Table 1.** Predicted responses to inbreeding under various fitness-selection scenarios.

selection on trait	expected inbreeding depression	rationale
directional	high	generates directional dominance (tends to fix beneficial alleles, removes dominant deleterious alleles, deleterious recessives remain) [32].
stabilizing	low/none	no directional dominance (mutations moving traits up and down are selectively equivalent) [32].
weak/neutral	low/none	traits weakly linked to fitness should not accumulate directional dominance and therefore show limited inbreeding depression [30].

**Figure 1.**
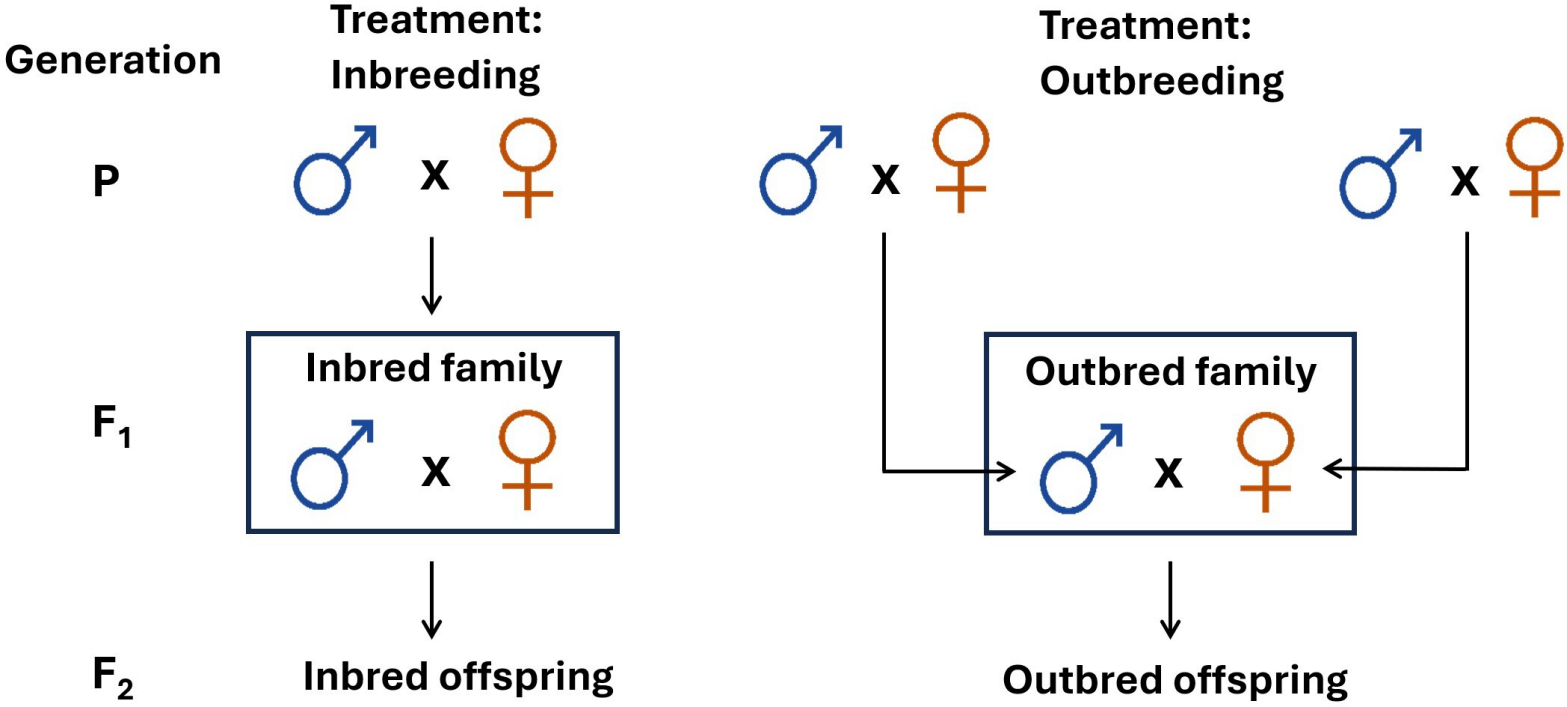
The experimental design used to generate inbred and outbred beetles. Virgin stock population beetles were randomly paired to form the parental generation (P). These then produced F1 offspring, which were randomly allocated into inbreeding or outbreeding treatments. F2 inbred offspring were obtained through F1 brother–sister matings (theoretical *F* = 0.25). Outbred F2 offspring were obtained through crossing unrelated males and females originated from different parental beetle pairs. In total, 120 out of 135 inbred families produced F2 inbred offspring. In total, 123 out of 136 outbred families produced F2 outbred offspring. Three F2 male offspring were collected per F1 family and subsequently used in analyses for a final sample size of 357 inbred and 369 outbred individuals from 120 inbred and 123 outbred families, although precise numbers in analyses varied if traits were damaged during dissection.

Here, we assess the static allometry of the sexually selected mandibles of the flour beetle *Gnatocerus cornutus* and the likely shape of selection acting on them. The mandible is a male-limited trait (females do not develop this trait: see figure 1 [34]) that readily responds to selection, and larger mandibles are favoured during male–male competition [35,36]. Additionally, when mandibles are enlarged through artificial selection, males gain an advantage during male–male competition and have greater mating success [34]. However, because of the suite of intersexual genetic correlations, the daughters of males with larger mandibles have lower reproductive success—basically their bodies are masculinized and they lay fewer eggs [37,38]. Furthermore, female choice tends to favour males with smaller mandibles, even though females may not always be able to exercise that choice [39], and females have lower fitness when mating with males having larger mandibles [40]. Thus, it appears the mandibles are under stabilizing selection, despite previous estimates of mandible scaling suggesting they are positively allometric [41]. Here, we reassess the allometry of beetle mandibles and use inbreeding to further assess the likely shape of historical selection acting on mandible size. Finally, because these beetles have rather recently moved into a new environment (stored products: [42]), which is now their primary habitat (and which we can replicate in the laboratory), employing inbreeding was a good way to infer past selection, although we acknowledge that what we document here could be a response to selection in this ‘new’ habitat. In any case, we find that despite strong positive allometry, mandibles appear to be under stabilizing selection.

## 2. Material and methods

### 2.1. Stock population

The *G. cornutus* beetle culture originated from adults collected in Miyazaki city, Japan, in 1957 [43] and has subsequently been maintained in the laboratory of the National Food Research Institute, Japan. The beetles used in this study were derived from that culture. They have been reared on 90% organic white flour (Doves Farm Foods) enriched with 10% brewer’s yeast (Brewer’s yeast; Thermo Fisher Scientific). Both rearing and experimentation were conducted in climate chambers maintained at 27℃, 60% relative humidity and with a photoperiod of 14 h : 10 h, light to dark cycle. These laboratory conditions mimic the natural conditions where *G. cornutus* was found [44].

At each generation, 100 males and females were reared on 400 g of food in a plastic pot (Nalgene 1000 ml; Thermo Fisher Scientific) for 4 weeks to allow for mating and oviposition. The food was then transferred to a plastic box (4 l), and additional food was added to bring the height of food in the box up to 20 mm. After approximately 10 days, 450 third instar larvae were sieved from the food and individually allocated to a single cell (1.5 × 1 cm) of a 24-well plastic plate. *Gnatocerus cornutus* does not pupate under high larval density [45], so isolating larvae from the culture ensures pupation and also ensures that the beetles do not interact with conspecifics immediately after eclosion. The larvae took approximately 10 days from the date of collection to pupate and eclose. Adult beetles (100 males and females) were then randomly collected to form the parents for the next generation.

### 2.2. Experimental design

We employed one generation of inbreeding to produce inbred beetles (figure 1). To obtain adult beetles, we randomly collected 1440 third instar larvae from the stock to set up the parental generation (P) and individually placed them in a cell of 24-well plastic plates (24 larvae per plate = 60 plates in total) without any food. Importantly, the beetles acquire the resources needed for development during the larval period and do not require food during pupation. After pupation, the pupae were observed daily for eclosion, and as soon as the adults emerged, they were collected and separated by sex into plastic pots (multi-purpose container, 250 ml, 78 × 70 mm; Sarstedt) with 75 g food (as above). Hence, the beetles had access to ad libitum food throughout the larval and adult stages (i.e. there was no nutritional limitation).

*Gnatocerus cornutus* takes up to 7 days to attain sexual maturity after eclosion [44,46], so monogamous pairs were set up 7 days after the last beetle emerged. Beetles from all of the pots were pooled (and kept separated by sex) and haphazardly paired in plastic cups (Majestic 4oz Clear Plastic Containers & Lids, CupsDirect) with 25 g food to make 450 parental beetle pairs. The same methods used for P generation were employed in subsequent generations (i.e. F1 and F2) unless stated otherwise.

Pairs were kept in the cups for 2 months, and 407 out of 450 pairs produced enough offspring for the F1 generation (figure 1); 1/3 of the parental beetle pairs (*n* = 135) were allocated to inbreeding treatment, which subsequently formed 135 F1 inbred families. The remaining pairs (*n* = 272) were allocated to outbreeding treatment. Here, one male offspring per pair was obtained from half of the pairs (*n* = 136), and one female offspring per pair was obtained from the another half of the pairs (*n* = 136). Crossing of those offspring established 136 F1 outbred families (figure 1).

Subsequently, 18 third instar larvae were collected per P-generation pair (to form the F1 animals) and reared individually until eclosion in the 24-well plastic plates. Emerging adults were separated by sex and housed in single-sex family groups in plastic vials (Drosophila vials, 25 × 93 mm; Genesee Scientific) with 7.5 g food until all beetles reached sexual maturity. For the inbreeding treatment, one male and one female per family were randomly selected per plate, and these brother–sister combinations were paired (theoretical *F* = 0.25) to generate inbred offspring (figure 1). For the outbreeding treatment, one male OR one female was randomly chosen per family, and these were paired with unrelated opposite-sex adults originated to produce outbred offspring (figure 1). All pairs were kept in the plastic cups with 25 g of food for two months.

After two months, 24 third instar larvae from each F1 pairing (family) were randomly collected and individually housed in 24-well plastic plates until eclosion and reaching sexual maturity. In total, 120 out of 135 inbred families produced F2 inbred offspring, and 123 out of 136 outbred families produced F2 outbred offspring. Three F2 males were haphazardly collected from each family and frozen at −20℃ for further analyses. Two inbred families produced fewer than three males, but they were also included in the analyses. In total, we collected 357 and 369 individual beetles for inbreeding and outbreeding treatments, respectively.

### 2.3. Imaging and linear measurements

**Figure 2.**
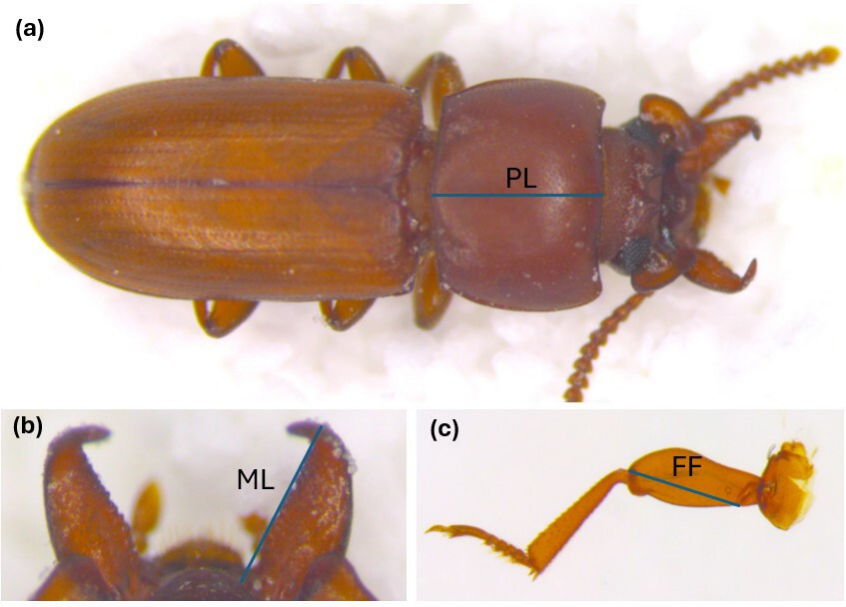
The *G. cornutus* traits measured as part of this study. (a) a dorsal image of a male, (b) mandibles, (c) a foreleg. Linear measurements were taken for PL (pronotum length), ML (mandible length) and FF (foreleg femur length).

Dorsal images of the beetles were taken using a microscope (Leica M125) with a mounted digital camera (Leica DFC 295). Specimens were carefully positioned on plastic weighing boats filled with fine white sand under the microscope. Images of male mandibles were taken separately to make sure they were in focus. After the imaging, the legs were dissected in drops of 50 : 50 glycerol and lactic acid using forceps under a dissecting microscope. Following dissection, the legs were mounted on cover glasses and covered with cover slips, and then images were taken of each leg. Image J (v. 1.53 t, National Institutes of Health, USA) was used to take linear measurements of the following traits in males: mandible, pronotum length, femur of forelegs (figure 2). These measures are used as our proxy for total trait length and have been used extensively in the study of this beetle (e.g. [35]), but we note that they largely ignore trait curvature. To assess measurement error, we measured 25 images of each trait twice. The consistency of measurements was evaluated using the intraclass correlation coefficient (package ‘irr’ in R: [47]). Each of the measurements was highly repeatable (mandible length = 0.984, 95% CIs: 0.964–0.993; pronotum length = 0.981, 95% CIs: 0.958–0.992; foreleg femur length = 0.986, 95% CIs: 0.969–0.994).

### 2.4. Statistical analysis

All statistical analyses were carried out using R (v4.3.0; [48]) within RStudio Desktop IDE (*v*2024.12.0.467; [49]). First, average values of three males per family were taken for each trait. Then, these data were checked for normality assumptions and outliers. The effect of inbreeding on the mandible, pronotum and foreleg femur length (hereafter leg length) was assessed using analysis of variance with treatment (inbreeding and outbreeding) as a predictor and family average trait values as dependent variables. Trait size usually correlates with body size [15], and body size itself can suffer from inbreeding depression (e.g. [50–52]), so we also tested whether the effect of inbreeding on the traits was independent of changes in body size. Previous studies employed leg length as an index of body size [26,53], so we used Pearson’s product-moment correlation to test for associations between leg length and body mass, another commonly used index of body size. The correlation was very high (*r*_51_ = 0.903, *p* < 0.001), so we decided to use the leg length as a proxy for body size. This was primarily because pronotum size (which is often used as a measure of size in beetles) is highly correlated (genetically integrated) with mandible size (see [34,35]). Additionally, selecting mandible size generates a strongly correlated response with pronotum size (e.g. [34]). As a result, we felt we needed a body size measure on the same measurement scale (i.e. length, not mass/volume) as our traits of interest, which was a more independent measure of body size, so that any inbreeding effects are not automatically confounded by the integrated nature of the mandibles and pronotum. To be confident that leg size reflects body size, we tested for leg-mass associations, and we found them to be strongly correlated (see above). Therefore, leg length seems to be a good structural measure of size. We note that leg length is often used as a measure of insect size (see, e.g. [54,55]), including in studies of allometry (e.g. [56]). Subsequently, analysis of covariance (ANCOVA) was performed to test the effect of inbreeding on the mandible and pronotum length by controlling for body size. Treatment was a predictor, leg length (body size) was a covariate and family average trait values were dependent variables.

Next, trait values of individual beetles (and family averages: data not shown) were used to quantify allometries of the mandible and pronotum length. There is some confusion over whether allometry should be quantified by fitting linear models on log-transformed data or fitting nonlinear models on untransformed data using power functions [57–60]. The former method is most widely applied since (i) log transformation enables comparison of traits with very different scales, and (ii) biological interpretation is more accessible [16,61–63]. Hence, we regressed log_10_-transformed trait values against log_10_-transformed body size (leg length). Also, prior to the analysis, the data were checked for outliers using generalized extreme studentized deviate (package ‘EnvStats’ in R: [64]), and six outliers were detected and removed. This method allows for the detection of multiple outliers while accounting for multiple testing [65].

Another question in the study of allometry is the appropriate line-fitting method [66–74]. Here, we use three methods to estimate slopes: ordinary least squares (OLS) regression, major axis (MA) and standardized major axis (SMA, also known as reduced MA). OLS regression assumes that the *X* variable (body size) is measured without error, and hence it tends to underestimate slopes [15,71]. MA and SMA (type II regressions) allow for measurement error in both *X* and *Y* variables, and it has been argued that these approaches are more suitable methods for the study of allometry [15,68,75]. We present results from all methods, but base our interpretation on type II outputs.

We extracted slopes and intercepts for each trait (the mandible and pronotum length) and for each treatment (inbreeding and outbreeding) using all three methods of line fitting, and subsequent tests were performed on these data. First, we tested whether the traits showed isometry (*β* = 1) using a one-sample test of slopes. Second, we tested whether the lines from two treatments shared a common slope and elevation for each of the traits, using ANCOVA for OLS (treatment as a predictor, body size as a covariate and an interaction term between the predictor and the covariate; see Zar [76] and Bartlett corrected maximum likelihood tests for MA and SMA [77]. These tests essentially ask if the slopes and intercepts of two treatments are different from each other (i.e. whether inbreeding had any effect on allometry). All tests on allometries were conducted using the package ‘smatr’ in R [77], except the test for common slope and elevation for OLS, which did not require that package. Where tests were conducted using ‘smatr’, we applied a more robust method known as Huber’s M estimation to acquire estimates, which was less sensitive to outliers [78].

All work was carried out under the approval of the Center for Ecology and Conservation’s Ethics Committee.

## 3. Results

### 3.1. Inbreeding depression in sexually and non-sexually selected traits

**Figure 3.**
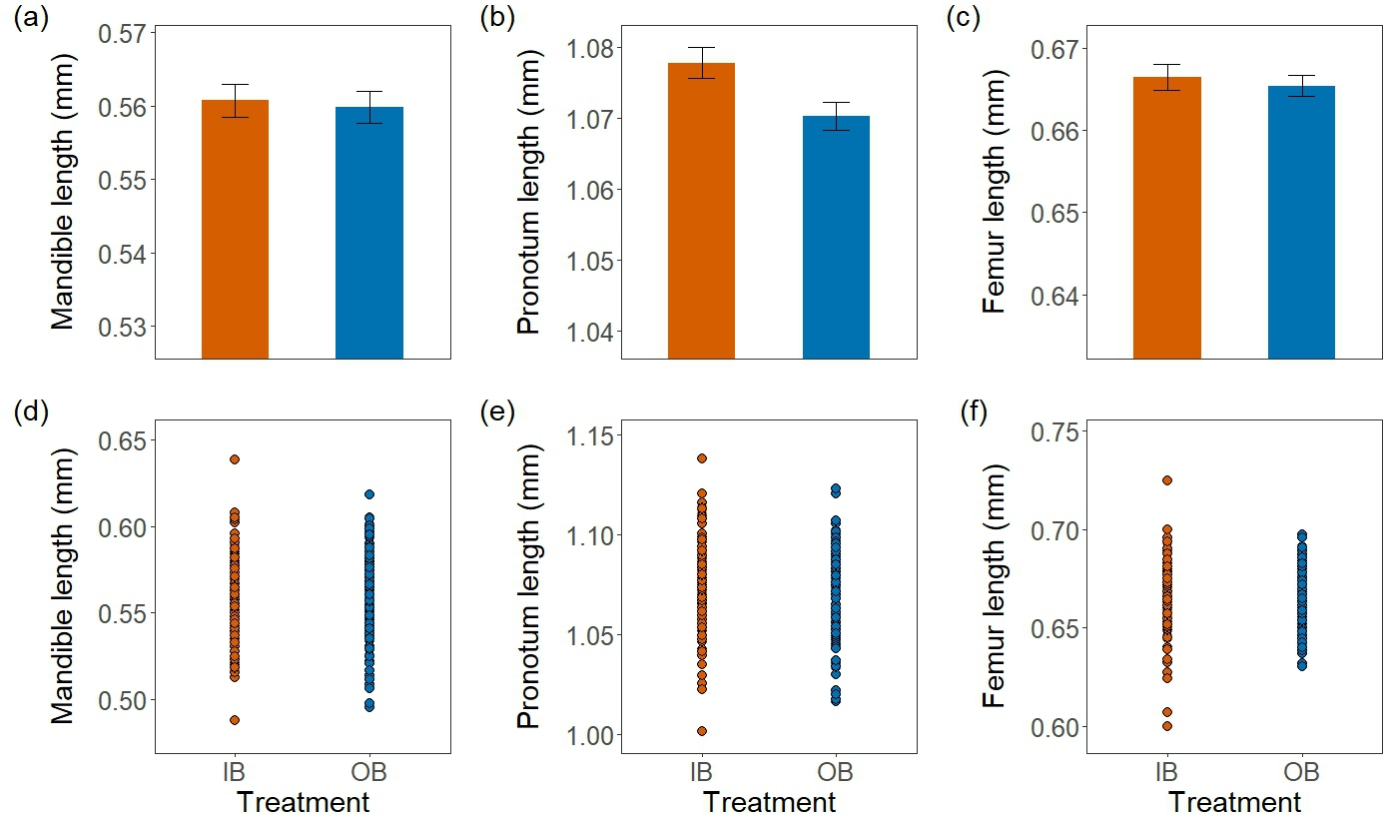
Top three plots illustrate mean mandible length (a), pronotum length (b) and foreleg femur length (c) of inbred (IB, orange bar) and outbred (OB, blue bar) treatments (± SE). There were no differences between the treatments for the mandibles and foreleg femur length, but there was a statistically significant difference between the treatments for the pronotum length. Bottom three scatter plots show the spread of data for mandible length (d), pronotum length (e) and foreleg femur length (f) of inbred (IB: orange dots) and outbred (OB: blue dots). Each dot represents a family mean. The data spread more in IB compared to OB in all traits.

We first tested for inbreeding depression in mandible size (sexually selected trait), pronotum length (non-sexually selected trait) and leg length (index of body size). There was no difference between the treatments in the mandible length (*F*_1, 241_ = 0.08, *p* = 0.78; figure 3a) nor in the leg length (*F*_1, 241_ = 0.25, *p* = 0.62; figure 3c), indicating no inbreeding depression in those traits. The mandibles also showed no inbreeding depression after controlling for body size (*F*_1,240_ = 0.13, *p* = 0.72). Conversely, there was a difference in the pronotum length between the treatments (*F*_1, 241_ = 6.29, *p* = 0.01; figure 3b), consistent with inbreeding depression in this trait. When controlled for body size, the difference in the pronotum length became highly significant (*F*_1,240_ = 17.75, *p* < 0.001).

### 3.2. Allometries of sexually and non-sexually selected traits

The mandible and pronotum length allometric slopes varied with the line-fitting methods (MA, SMA, OLS; table 2). First, we tested whether allometries of sexually and non-sexually selected traits were isometric (i.e. *β* = 1) in each treatment. Mandible slopes were greater than 1 in both inbreeding (IB) and outbreeding (OB) treatments when MA and SMA lines were fitted (table 2; IB_MA/SMA_: *F*_1, 352_ = 236.04, *p* < 0.001; OB_MA/SMA_: *F*_1, 364_ = 247.45, *p* < 0.001). However, the OLS fitted slopes for the mandibles were not significantly greater than 1 (table 2; IB_OLS_: *F*_1, 352_ = 1.43, *p* = 0.23; OB_OLS_: *F*_1, 364_ = 0.99, *p* = 0.32) and were lower than the MA or SMA estimates as expected. The pronotum length slopes were not different from 1 for MA and SMA fitted lines (table 2; IB_MA/SMA_: *F*_1, 352_ = 0.66, *p* = 0. 42; OB_MA/SMA_: *F*_1, 364_ = 0.05, *p* = 0.83), but again, the OLS slope estimates showed negative allometry in both treatments (table 2; IB_OLS_: *F*_1, 352_ = 44.74, *p* < 0.001; OB_OLS_: *F*_1, 364_ = 30.80, *p* < 0.001).

**Table 2.** Estimates of allometric relationships of mandible (sexually selected trait) and pronotum length (non-sexually selected trait) regressed against body size (leg length). The estimates were obtained from three methods of line fitting: MA, SMA and OLS regression. For each treatment and trait, multiple *r*^2^ was the same across all methods. *P*_0_ values are from the tests of slopes against zero, and *P*_1_ values are from the tests of slopes against one (i.e. isometry).

allometry of mandible and pronotum length												
treatment	trait	*N*	method	multiple *r*^2^	intercept	95% CI for intercept lower CI upper CI		slope	95 % CI for slope lower CI upper CI		*P*_0_	*P*_1_
inbreeding	mandible	354	MA	0.293	−5.687	−7.119	−4.255	2.987	2.546	3.587	<0.001	<0.001
			SMA		−2.869	−3.403	−2.334	1.989	1.809	2.187	<0.001	<0.001
			OLS		−0.220	−0.704	0.263	1.051	0.880	1.223	<0.001	0.233
	pronotum length	354	MA	0.538	0.312	0.066	0.557	0.963	0.880	1.055	<0.001	0.417
			SMA		0.290	0.095	0.485	0.971	0.904	1.043	<0.001	0.417
			OLS		1.179	0.999	1.359	0.656	0.593	0.720	<0.001	<0.001
outbreeding	mandible	366	MA	0.309	−6.249	−7.865	−4.633	3.187	2.694	3.873	<0.001	<0.001
			SMA		−3.010	−3.568	−2.452	2.040	1.852	2.247	<0.001	<0.001
			OLS		−0.592	−1.107	−0.078	1.183	1.001	1.365	<0.001	0.321
	pronotum length	366	MA	0.692	0.229	0.021	0.438	0.992	0.921	1.069	<0.001	0.830
			SMA		0.225	0.052	0.399	0.993	0.934	1.057	<0.001	0.830
			OLS		0.615	0.449	0.781	0.855	0.796	0.914	<0.001	<0.001

**Figure 4.**
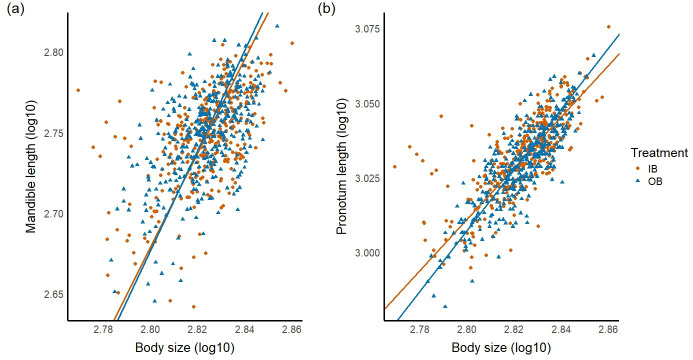
Allometry of (a) the mandibles and (b) the pronotum length for both IB (inbreeding, orange circle) and OB (outbreeding, blue triangle) treatments. Body size (= leg length, *x*-axis), the mandible and pronotum length (*y*-axis) are log_10_-transformed values. MA lines were fitted through the data points (IB in orange line, OB in blue line). The slopes were steeper for the mandibles (positive allometry) compared to the pronotum length (isometry). The slopes were slightly steeper for OB in both traits, but there were no statistically significant differences. The intercepts were different between the treatments for the pronotum length, but not for the mandibles. Using family means rather than individual-level data produced qualitatively similar results (see electronic supplementary material).

Next, we investigated whether the slopes and intercepts of allometries were different between inbreeding and outbreeding treatments. Both treatments shared a common slope for the mandible length for all line-fitting methods (MA: lr = 0.27, *p* = 0.61, *β*_com_ = 3.09, figure 4a; SMA: lr = 0.13, *p* = 0.72, *β*_com_ = 2.01; OLS: *F*_1, 716_ = 1.07, *p =* 0.30), indicating there was no difference in trait scaling between the treatments. Also, both treatments shared a common elevation for the mandible length in all line-fitting methods (MA: *W* = 0.76, *p* = 0.38, *α*_com_ = −5.93, figure 4a; SMA: *W* = 0.41, *p =* 0.52, *α*_com_ = −2.94; OLS: *F*_1, 716_ = 0.05, *p* = 0.83), indicating there was no difference in the intercepts either. Hence, inbreeding had no effect on the allometry of the mandibles.

For the allometry of pronotum length, both treatments shared a common slope (i.e. no difference in the slopes) according to the tests on MA and SMA lines (MA: lr = 0.24, *p* = 0.63, *β*_com_ = 0.98, figure 4b; SMA: lr = 0.22, *p* = 0.64, *β*_com_ = 0.98). However, the test on OLS lines showed that the slopes were different between the treatments (OLS: *F*_1, 716_ = 20.13, *p* < 0.001). Furthermore, treatments did not share a common elevation for the pronotum length for all line-fitting methods (MA: *W* = 9.79, *p* < 0.01, *α*_com_ = 0.25, figure 4b; SMA: *W* = 9.73, *p* < 0.01, *α*_com_ = 0.25; OLS: *F*_1,716_ = 22.33, *p* < 0.001), indicating that the intercepts differed between the treatments, with the intercept being greater across inbred individuals. Hence, inbreeding had an effect on the intercept of the pronotum scaling relationship.

## 4. Discussion

The positive allometry of sexually selected traits has been noted as one of the least well-understood patterns in animal form [4], but it has been suggested that directional selection on trait size is critical for the evolution of positive allometry (reviewed in [15]). Here, we found no evidence for inbreeding depression in mandible size, consistent with stabilizing selection on this trait, and yet mandibles are positively allometric using the most widely agreed methods for estimating trait scaling.

A lack of inbreeding depression in mandible size could conceivably be because mandibles are not linked to fitness [32]. However, this is clearly not the case (e.g. [34–36]). It is also possible that the inbreeding we used here was not severe enough to cause inbreeding depression in morphology and that longer durations of inbreeding would cause declines in mandible size, thus providing evidence of directional selection [32]. Multi-generation durations of inbreeding are occasionally required to generate inbreeding depression in some sexually selected traits (e.g. ejaculates: [79]), with suggestions that these characters have an inbreeding threshold that needs to be passed before effects are seen [80]. This is possible and something we need to explore further; however, another beetle character (pronotum length) showed inbreeding effects with the level of inbreeding we applied (as did elytra length—unpublished data), suggesting full-sib mating (*f* = 0.25) is sufficient to impact morphology. Furthermore, this level of inbreeding has impacts on male traits in a closely related beetle [27], and sexually selected traits generally show inbreeding depression after one generation of sib-sib mating [81] (further generations of inbreeding also run the risk of purging—the selective loss of deleterious alleles [82]). Previous evidence points to stabilizing selection on the mandibles, as male–male competition favours larger mandibles, and this is counter-balanced by sexually antagonistic effects on females, by female mate choice [34,37,39], and additionally, females have lower reproductive success when mating with males having large mandibles [40]. This agrees with our inbreeding findings, and so currently, the balance of evidence suggests stabilizing selection on mandible size—although mandibles are enlarged and presumably were subject to directional selection at some time in the past.

Despite apparently being subject to stabilizing selection (see discussion in [32]), mandibles show positive allometry (but, for example, pronotum length does not) when estimating slopes with type II regression. This is interesting because formal theory and verbal argument both suggest directional selection on trait size is required to generate positive allometry [4,14,15,19]. Our data suggest this need not be true. Theory does indicate that minor changes in fitness functions and the relationships between traits can generate a range of allometries [4,14,19], and so reconciling stabilizing selection and positive allometry may be possible, especially if selection favoured smaller body size. The increased pronotum width in inbred animals hints that this could be the case, but our measure of size, leg length, showed no changes with inbreeding. It is also possible that historical directional selection on mandibles (mandibles are enlarged after all) resulted in positive allometry, and this scaling relationship remains despite stabilizing selection currently and in the recent past. This possibility would be consistent with studies showing that directly selecting on allometry does not alter allometric slopes, but instead alters the intercept of the trait-size relationship (e.g. [83]). This is also what was found when mandible size was subject to directional selection in the flour beetle—the intercept evolved but not the slope [35], also see [41]—although in the current study we found no inbreeding depression for the mandible-size intercept. Nonetheless, if this were the explanation for the mandible data (no change in allometry despite a change in selection), it seems to contrast with theoretical findings indicating allometric slopes can evolve readily when selection changes [4,19]. In any case, male beetles with smaller mandibles can secure matings [39], which is an important condition facilitating the evolution of positive allometry in the most general model of allometry evolution [4]. Overall, however, our data challenge theory by suggesting that either positive allometry does not require directional selection or that, despite changes in selection, allometry does not evolve readily.

There was also no significant inbreeding depression for the allometric slope of the mandible-size relationship, which suggests no directional selection on allometry itself, and as noted, previous work suggests the slope of the mandible-size relationship does not respond readily to direct selection [35]. However, the positive allometry we document for mandible size is consistent with studies of weapon allometry generally [14,16] and with previous work on this beetle [35]. The allometry of pronotum length only serves to highlight the ‘special’ scaling of mandibles. Interestingly, there was some indication of inbreeding impacts on pronotum allometry and strong evidence that inbreeding altered the scaling intercept of the pronotum-size relationship. This suggests that inbred individuals invest more in pronotum size at mean size (as slope differences between treatments were not always detected) and reflect other findings showing slope-intercepts are less constrained than slopes (e.g. [83]). Additionally, inbreeding led to a larger rather than smaller pronotum size. This was unexpected as high fitness males tend to have longer pronotums [35], and hence inbred males were expected to have shorter pronotums. How selection on females impacts this expectation requires further investigation, but there is selection for smaller pronotum size in females and strong intersexual genetic correlations for body size and shape [35,36,38,84]. So it is possible that there is net selection for smaller pronotum size, and hence inbreeding results in increased size.

The arguments above are predicated on type II regression being the best way to estimate trait scaling. This is a standard approach in studies of allometry (e.g. [10,20]) and is recommended because least squares underestimates true slopes because it assigns all measurement error to the dependent variable, which is clearly false [15,68,85]. Our arguments also assume the level of inbreeding we used was sufficient. We note again that we documented inbreeding depression for one trait, and for mandibles, we did not expect inbreeding depression because all the available evidence is indicative of stabilizing selection on this character, which would preclude inbreeding depression for mandible size [32]. Hence, we think the approach and methods we employed were appropriate.

Overall, and assuming the experimental intensity of inbreeding was sufficient, we are left with two interpretations of our findings: either directional selection is not required for positive allometry, or there is significant inertia in the evolution of trait scaling such that even when the form of selection changes, allometry does not. In either case, the beetle data suggest the evolution of trait scaling is more nuanced than currently thought.

## Data Availability

Supplementary material is available online [86].
